# New approaches to diagnose and target reproductive failure in cattle

**DOI:** 10.1590/1984-3143-AR2020-0057

**Published:** 2020-09-15

**Authors:** Ky Garrett Pohler, Sydney Taylor Reese, Gessica Araujo Franco, Ramiro Vander Oliveira, Rafael Paiva, Lohana Fernandez, Gabriela de Melo, José Luiz Moraes Vasconcelos, Reinaldo Cooke, Rebecca Kyle Poole

**Affiliations:** 1 Department of Animal Science, Pregnancy and Developmental Programming Area of Excellence, Texas A&M University, College Station, TX, United States; 2 Departamento de Produção Animal, Universidade Estadual Paulista (UNESP), Botucatu, SP, Brasil

**Keywords:** pregnancy, bovine, embryonic mortality, pregnancy loss, pregnancy detection

## Abstract

Reproductive failure and pregnancy loss in cattle are some of the largest economic burdens to cattle producers and one of most perplexing factors influencing management decisions. Pregnancy loss may occur at any point during gestation with the largest percentage of loss occurring in the first 30 days and, subsequently, decreasing as the pregnancy progresses. Losses may be attributed to numerous factors, predisposed issues or environmental conditions such as nutritional stressors or disease. From a research perspective, determining the exact causes of pregnancy loss or embryonic mortality in cattle have been difficult, due to limitations of accurately determining early gestation pregnancy status. Until methods that precisely determine embryo success early in gestation are available, our understanding of *in vivo* pregnancy loss will lack clarity necessary to develop management strategies to decrease such loss. In this review, we will briefly discuss the pivotal periods of pregnancy loss affecting beef and dairy cattle, methods and technologies to determine pregnancy status and embryo viability and potential opportunities to decrease reproductive failure.

## Introduction

**Figure 1:**
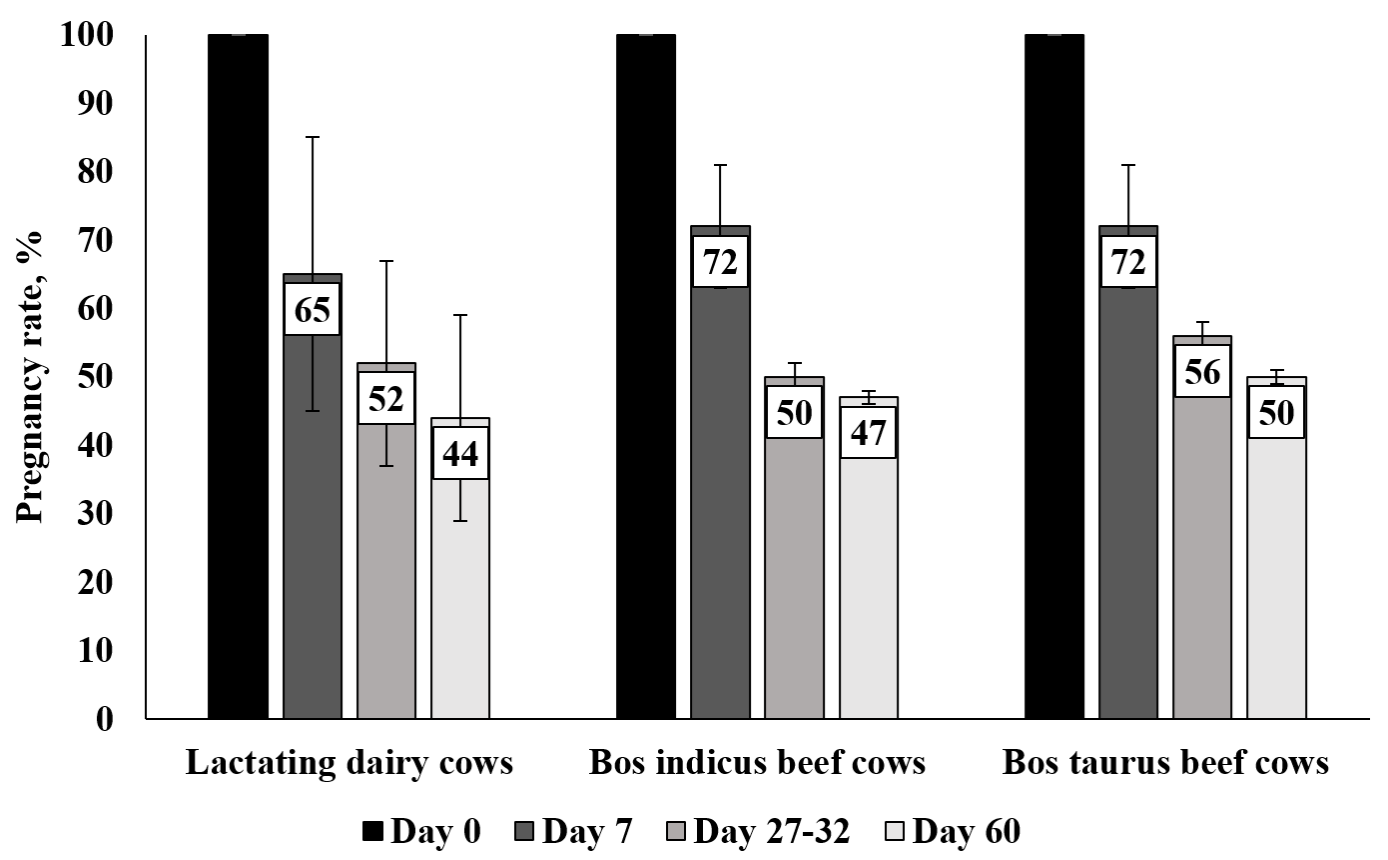
Summary of reproductive success during first two months of gestation in lactating dairy cows (111) and beef cows (112). Losses between day of breeding (Day 0) and day 7 includes fertilization failure as well as early embryonic development failure. Values are represented as estimated mean (bar) and variance encountered in studies (error bar lines).

Embryonic mortality and pregnancy loss remain major issues in domestic livestock production. In beef and dairy cattle, reproductive inefficiency has been reported to cost global producers over $1 billion dollars annually (USDA, 2009, 2010). While research and our understanding have drastically increased around the area of reproductive failure, embryonic mortality, and pregnancy loss, we have been limited by the inability to accurately determine timing and viability of embryos/fetuses *in vivo*, specifically during early gestation. In the last 4 years, two systematic reviews have been conducted in beef and dairy summarizing critical periods of pregnancy loss identified by currently available technology to determine embryonic/fetal presence (Wiltbank et al., 2016; Reese et al., 2020); summarized in Figure 1.

Timing of embryonic and fetal mortality in cattle can vary substantially based on production status, genetic composition, environment and management conditions. Both dairy and beef cattle have relatively high fertilization rates, with a majority of embryos still viable 7 days after insemination. At day 7, 70-75% of beef females and ~ 65-70% of dairy females that are artificially inseminated (AI) after estrus expression or bred by fixed timed AI will have a developing blastocyst (Wiltbank et al., 2016; Reese et al., 2020). Despite these relatively high percentages, fertilization rates are negatively affected often by poor quality gametes, especially low-quality semen due to sire infertility or improper semen handling. Animals that fail to establish pregnancy during this initial period are difficult to detect as they appear to cycle normally. Unfortunately, harvesting the reproductive tract is one of the only accurate ways to determine embryonic presence during this time period which is constrained by cost, need for animal sacrifice and unknown success of the embryo through later stages of development.

The next 3 weeks of development are pivotal for successful establishment of pregnancy. Uterine environment is critical as the embryo transcends from the oviduct to the site of implantation. Asynchrony between the uterus and the embryo can be problematic as the uterine environment will not “wait” for an embryo, although an embryo can accelerate or decelerate its development to some degree (Pope, 1988). Historically, maternal recognition of pregnancy failure was believed to account for a significant percentage of pregnancy loss (Diskin and Sreenan, 1980; Roche et al., 1981). Research indicates that high producing dairy cattle are especially susceptible to pregnancy loss during this period (Lucy, 2001; Wiltbank et al., 2016). Without maintenance of the corpus luteum (CL) and sustained levels of progesterone (P4) as a result of interferon-tau (IFNT) secretion, luteolysis occurs and pregnancy cannot continue (Mann and Lamming, 1999). Pregnancy during this time period can be indirectly determined by the presence of Interferon-stimulated genes (ISG), which unfortunately have high levels of false positives (Green et al., 2010; Melo et al., 2020a). In dairy cattle, pregnancy loss between day 19 and 27 is reported as approximately 20% (Wiltbank et al., 2016) while in beef about 15% between day 16 and 32 (Reese et al., 2020). Detection of the embryonic heartbeat between day 28 and 32 of gestation implies the end of the early embryonic period and transition into late embryonic development. During the first month of gestation, embryonic mortality or pregnancy loss affects 44 to 50% of pregnancies in both beef and dairy cattle (Wiltbank et al., 2016; Reese et al., 2020). Interestingly, in beef cattle, cows with *Bos indicus* influence (at least 3/8 *Bos indicus* influence) seem to have increased reproductive failure during this developmental period (Reese et al., 2020).

Late embryonic and early fetal mortality make up a small percentage of overall pregnancy losses; however, due to management practices, many go undetected and result in greater economic defects. It is estimated that late embryonic mortality affects 5 to 8% of beef cattle pregnancies while upwards of 15% of dairy pregnancies may be terminated during this period (Wiltbank et al., 2016; Reese et al., 2020). Causes of late embryonic mortality are less understood as research has focused primarily on examining the contributing factors to early embryonic loss. During late embryonic development, hallmark placentome formation and exponential placental expansion occur (Assis et al., 2010). Deficiencies in placental growth or function can have severe consequences and may be an overlooked factor of pregnancy loss.

**Figure 2:**
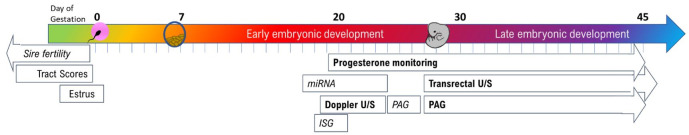
Advancements in detecting and managing pregnancy loss through early gestation. Established methods for detection of pregnancy loss are indicated in **BOLD**; methods that are currently the focus of research are indicated in *italics*.

Simply knowing when pregnancy loss or embryonic mortality occurs is only part of the story. From a scientific perspective, the ability to understand and determine the causes of loss are critical to develop technology to overcome and prevent such losses. Therefore, a critical need exists to develop an ideal pregnancy test or series of tests to determine the presence of an embryo and its potential viability. From a production point of view, an ideal pregnancy test should have high sensitivity (i.e., correctly identify pregnant animals), high specificity (i.e., correctly identify non pregnant animals), and be simple and inexpensive to conduct under field conditions. For scientific purposes, tests that measure embryonic or fetal viability are important to further research in this area. Currently, there are three categories of embryonic or pregnancy detection tests: manual, chemical pregnancy specific and chemical non-pregnancy specific. Manual methods use physical palpation or visualization to identify pregnant animals. Pregnancy specific or embryonic specific tests use markers that are directly produced by the conceptus or developing pregnancy while non-pregnancy specific tests rely on indirect measurements. In addition, it is important to evaluate the parental (maternal and paternal) contributions to likelihood of pregnancy loss throughout embryonic development. The purpose of this review is to highlight different methods to target or reduce reproductive failure in cattle; summarized in Figure 2.

## Manual detection methods

### Rectal palpation and ultrasonography

As the conceptus develops during early gestation, fluid accumulates within the allantoic and amniotic cavities making rectal palpation a common method of pregnancy diagnosis in cattle. Experienced technicians can detect pregnancy as early as day 30 to 35 of gestation by palpation of the amniotic vesicle (Roberts, 1956; Momont, 1990); however, this method of pregnancy diagnosis is more accurately utilized 40 to 60 days after insemination when there is greater fluid accumulation and to avoid puncturing the amniotic vesicle and terminating the pregnancy (Ball and Carroll, 1963). While rectal palpation is reliable and a widely accepted industry method for pregnancy diagnosis, it is difficult to assess embryonic viability and/or detect pregnancy loss with this technique. Transrectal ultrasonography (US) allows for earlier detection of pregnancy, as early as day 26 to 29 of gestation, and for morphological assessment of the uterus, ovaries, and real time embryonic/fetal viability (via heartbeat detection) in cattle (Pierson and Ginther, 1984; Curran et al., 1986; Kastelic et al., 1988; Beal et al., 1992). Due to the ability to visualize the pregnancy, US is considered the gold standard of pregnancy diagnosis with accuracy nearing 100% when conducted by experienced technicians (Romano et al., 2006). Regarding pregnancy loss detection, spontaneous embryonic mortality occurring between day 24 to 40 of gestation can be diagnosed via US by the absence of a heartbeat, placental detachment, or reduced placental fluid volume (Pierson and Ginther, 1984; Curran et al., 1986; Kastelic et al., 1988).

**Figure 3:**
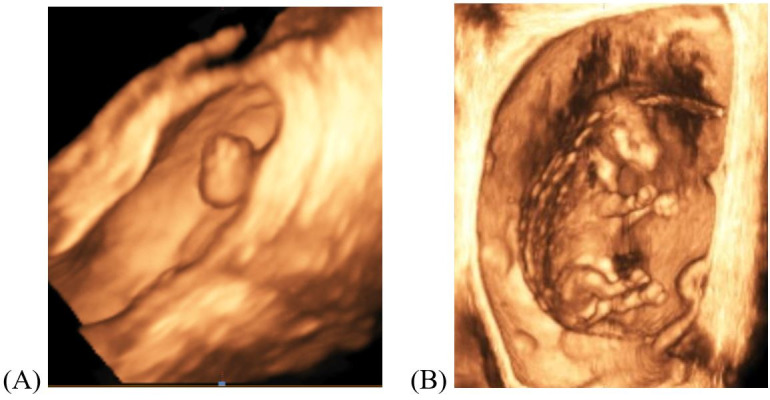
Day 32 (A) and 60 (B) bovine embryo/fetus imaged with 3D/4D technology on a Samsung HS60 ultrasound (Pohler lab).

Three-dimensional/four-dimensional (3D/4D) US has been widely used in human medicine to visualize pregnancy and assess the viability of the developing fetus (Hata et al., 1997). Currently, adoption of this technology for use in large animals is limited due to high costs of the necessary equipment and operational difficulties. During early embryonic development, the bovine embryo lacks definition to properly capture a 3D/4D image. By day 45 of gestation, development of anatomical structures (e.g. head, body, legs; Kähn, 1989) is sufficient to capture a 3D/4D image (Figure 3); however, the fetus must be properly oriented within the uterus and, thus, requires advanced technical skills. Given that fetal mortality (≥ day 45 of gestation) accounts for a low percentage of the overall pregnancy loss in cattle (Santos et al., 2004; Reese et al., 2020), use of 3D/4D US may not provide sufficient or beneficial information for determining pregnancy status or pregnancy loss when compared to other available methods. Over the next few years, however, further research can determine the potential of using such technology.

## Doppler ultrasonography

The establishment and maintenance of pregnancy requires the presence of a functional CL and adequate P4 concentrations (Mann and Lamming, 1999; Parr et al., 2012). In non-pregnant females, between days 15 to 18 of the estrous cycle, P4 concentrations decrease in response to prostaglandin F2α (PGF2α) released by the uterus (Niswender et al., 2000), inducing the CL to regress (Ginther et al., 2010). Doppler US allows identification of structural luteolysis by visualization of decreasing CL blood perfusion during its regression, which can allow for the diagnosis of non-pregnant females earlier than traditional US methods (Herzog et al., 2010; Pugliesi et al., 2014; Scully et al., 2015). This method is efficient in ruminants because decreasing blood perfusion during CL regression correlates with decreasing P4 concentrations (Herzog et al., 2010; Balaro et al., 2017).

Doppler US has been used primarily in beef herds for pregnancy diagnosis between days 20 and 22 of gestation with accuracy exceeding 90% (Pugliesi et al., 2014, 2018; Motta et al., 2020). One of the main advantages of the early pregnancy diagnosis by Doppler US is its high sensitivity, close to 100%, which results in few false-negative diagnoses (Pugliesi et al., 2014; Melo et al., 2020a). This method, however, results in 15 to 20% false-positive diagnoses (Siqueira et al., 2013; Pugliesi et al., 2018; Melo et al., 2020a) that may be attributed to delayed ovulation in response to the synchronization protocol, extended estrous cycles (> 22 days) or early embryonic mortality before secondary diagnosis (Pugliesi et al., 2018). This may be exacerbated in dairy herds that have high incidence of early embryonic mortality between days 24 and 30 of gestation (Pohler et al., 2016a; Reese et al., 2018). Despite being a good predictor of pregnancy, Doppler US by itself is not efficient in monitoring the viability of the fetus or predicting pregnancy losses, since a reduction in circulating P4 concentrations occurs simultaneously or after embryonic/fetal death (Pohler et al., 2013, 2016a). When used in conjunction with other markers (see below), such as ISG or pregnancy associated glycoproteins (PAG), there is a 5 to 10% decrease in the false-positive diagnoses, which could contribute to identification of potential pregnancy losses (Melo et al., 2020a).

## Chemical-based methods: pregnancy specific

### Pregnancy associated glycoproteins

Pregnancy associated glycoproteins (PAG) are placental products produced by giant trophoblast cells (TGCs) of the trophectoderm. Around day 19 to 21 of gestation, TGCs migrate across the microvillar junction, fuse with the uterine epithelium and deliver products including placental lactogen and PAG into the uterine stroma that enter the maternal circulation (Wooding and Wathes, 1980; Wooding, 1982; Pohler et al., 2015). The first significant increase of PAG in maternal circulation can be identified between day 22 and 24 of gestation (Arnold et al., 2012; Pohler et al., 2013); however, physiological roles of PAG remain unclear. It has been hypothesized that PAG may help process growth factors at the placental-uterine interface, play a role in adhesion between the uterus and placenta, or act as a factor controlling maternal immune modulation (Austin et al., 1999; Hoeben et al., 1999; Wallace et al., 2015). Several factors have been shown to influence circulating PAG concentration, including parity, days post-partum, estrus response, sire and embryonic viability (Pohler et al., 2016b; Franco et al., 2018).

Over 20 separate PAG genes have been identified in the bovine genome, all pregnancy specific, which has contributed to the development of multiple commercialized pregnancy diagnostic tests for ruminants (Butler et al., 1982; Sousa et al., 2006). In addition to accurate pregnancy diagnosis, current research aims to understand the correlation between circulating PAG concentration and embryonic mortality. Several research groups have reported that circulating concentration of PAG can be used as a biomarker of pregnancy and embryonic viability (Silva et al., 2007; Romano and Larson, 2010; Pohler et al., 2016a, b). Across all classes of cattle, females that experience late embryonic loss have decreased circulating concentrations of PAG around day 30 of gestation compared to cows that maintain pregnancy until term (Pohler et al., 2013, 2016a, b). Pohler et al. (2016a) established a cutoff for PAG concentrations in serum samples on day 31 of gestation in dairy cows submitted to fixed-timed artificial insemination (FTAI) and timed embryo transfer, achieving 95% accuracy in predicting late embryonic mortality. Similar accuracy was achieved predicting embryo mortality in Nelore beef cows subjected to FTAI, where concentrations <0.72 ng/mL on day 28 of gestation resulted in pregnancy loss (Pohler et al., 2016b). Similar to AI pregnancies, Breukelman et al. (2012) reported cows that experienced pregnancy loss between days 26 and 100 after embryo transfer had decreased circulating concentrations of PAG. Overall, higher circulating PAG concentrations is directly correlated to a decreased incidence of embryonic loss (Szenci et al., 2000; Thompson et al., 2010). Surprisingly, there is no significant correlation between embryo size and circulating PAG concentrations on different days of gestation (Pohler et al., 2014; Pohler lab *Unpublished data*) suggesting that these pregnancy losses are not a consequence of delayed or underdeveloped embryo growth alone. Using a serial embryo transfer model comparing high and subfertility heifers, PAG concentrations were shown to be embryonically driven and not repetitive across multiple pregnancies in a single female (Reese et al., 2019). Even though subfertile heifers experienced greater pregnancy losses, circulating PAG concentrations associated with successful pregnancies did not differ between high and subfertile heifers. Similar to previous studies, incidences of late embryonic mortality were associated with decreased circulating PAG concentrations in both high and subfertility heifers (Reese et al., 2019). Despite the consistent observation of decreased PAG in pregnancies that undergo late embryonic mortality, cutoff prediction values are often lower than a majority of PAG concentrations in animals undergoing pregnancy loss. Breukelman et al. (2012) identified 4 unique PAG-1 profiles in cows undergoing pregnancy loss which may reflect the different causes of individual cases of pregnancy loss or timing of PAG expression and profiles in individual animals, thus increasing the challenge associated with predicting pregnancy loss. In addition, Gatea et al. (2018) reported significant antibody dependence in predicting pregnancy loss and showed differences across reproductive management techniques.

With the established usability of PAG at day 28 to 30 of gestation, PAG concentrations as early as day 22 have been evaluated as both a pregnancy diagnostic and embryo viability test. Compared to non-pregnant cows, pregnant cows and heifers have an increased circulating concentration of PAG by day 24 and 22 of gestation, respectively (Arnold et al., 2012; Martins et al., 2018; Reese et al., 2018; Middleton and Pursley, 2019; Oliveira et al., 2020), suggesting that PAG has the potential to be used as a biomarker for early pregnancy diagnosis in cattle. If applied properly, this advancement could identify non-pregnant animals and reduce the interbreeding/calving intervals by allowing earlier resynchronization and rebreeding. Although the detection of PAG is possible as early as day 24 of gestation, the benefits as a pregnancy diagnosis tool at this stage may be mitigated by the high incidence of embryonic mortality that occurs after day 24, increasing the number of false-positive diagnoses; however, this approach might allow investigation of a window of pregnancy loss previously unexplored. Embryonic mortality between days 24 and 30 may be overlooked because of the challenges historically associated with its identification; however, reports indicate that between 5 and 10% of pregnancies are lost during this period (Reese et al., 2018, 2020; Oliveira et al., 2020). In addition to the high rate of false positives, false negative diagnosis can be an issue as circulating PAG concentrations increase at different rates in individual cows. Reese et al. (2018) reported up to a 55% false negative rate using a 90% confidence cutoff value; whereas, Middleton and Pursley (2019), using a different antibody-assay combination, observed a 6% false negative rate indicating that antibody selection towards early secreted PAGs is critical. One challenge is day 24 PAG concentrations exhibit increased variability compared to day 30 samples, making it more difficult to establish cutoff values for pregnancy success or embryonic mortality predictions. Some studies show no difference in day 24 PAG concentrations between dairy females that undergo pregnancy loss and maintain pregnancy (Reese et al., 2018) while others identify differences in lactating dairy and beef cows (Middleton and Pursley, 2019; Oliveira et al., 2020). Discrepancies in these early PAG results may be partially attributed to the large family of PAG genes, differing in timing of PAG expression by the developing conceptus or antibody reactivity (Wallace et al., 2015; Gatea et al., 2018). Both in-house antibody combinations and commercially available diagnostic tests have been utilized in research adding to the confusion in these data. When circulating concentration of PAG at day 22 to 24 was analyzed to predict pregnancy success, different results were observed (Reese et al., 2018; Middleton and Pursley, 2019; Oliveira et al., 2020), indicating that assay refinement and studies with larger sample sizes are needed to improve the predictive value to an acceptable point for use in applied reproductive management.

## Chemical-based methods: non-pregnancy specific

### Progesterone

Detection of P4 is a non-pregnancy specific diagnosis method and is most commonly used in the dairy industry to assess CL regression and potential return to estrus following insemination (Nebel, 1988). The differences in P4 levels in either serum or milk between a pregnant and non-pregnant cow can be used as a biomarker for early pregnancy detection. Cows with low P4 levels (< 1 ng/mL) 18 to 24 days after insemination would be classified as non-pregnant, having undergone luteolysis, and cows with high P4 levels (≥ 1 ng/mL) during this period would be classified as potentially pregnant (Sasser and Ruder, 1987; Nebel, 1988; Niswender et al., 2000). This method can diagnose non-pregnant cows with an accuracy between 81 to 100%; however, the efficiency of positively diagnosing pregnancy varies between 60 and 100% (Nebel et al., 1987). False positive pregnancy diagnoses likely reflect cows with longer luteal phases (three vs. two follicular waves), a persistent CL, luteal or luteinized cysts, or cows that will undergo early embryonic mortality (Pohler et al., 2015). Therefore, non-specificity and consequential high prevalence of false positives has limited the use of this method as a marker of pregnancy or embryonic viability. More recent data using an on farm lateral flow immunochromatographic assay has reported promising results in identifying non-pregnant cows without a functional CL; however, had low specificity in diagnosing pregnancy (Omontese et al., 2020). Elevated concentrations of circulating P4 immediately post-insemination has been associated with conceptus elongation (Garrett et al., 1988; Carter et al., 2008) and increased pregnancy rates (Mann and Lamming, 1999; Stronge et al., 2005) in cattle. Regarding pregnancy loss detection, some discrepancies exist in the literature between the relationship of P4 and cows undergoing embryonic mortality. Starbuck et al. (2004) demonstrated that cows with < 3.76 ng/mL plasma P4 at week 5 of gestation were more likely to experience embryonic mortality than cows with a greater concentration of P4; however, a majority of cows (77%) in the low P4 group did not experience pregnancy loss. A later study demonstrated that serum P4 levels between day 28 and 30 in pregnancy cows was not predictive of pregnancy loss (Pohler et al., 2013).Therefore, elevated P4 levels between day 18 to 24 is most useful for determining pregnancy establishment and using relatively low P4 levels as a predictor for pregnancy loss is less reliable compared to other chemical-based methods discussed in this review.

### Interferon-tau stimulated genes

Maternal recognition of pregnancy in cattle occurs during the period of embryo elongation when IFNT is secreted by trophectoderm cells of the developing conceptus (Bazer et al., 1997; Gray et al., 2002) to prevent the release of PGF_2α_ by the endometrium, thus maintaining the CL function and P4 production (Bazer et al., 2015). In addition to its role in the process of maternal recognition of pregnancy, IFNT is also able to induce the expression of several ISG by the endometrium (Mirando et al., 1991) and reaches the peripheral circulation through the uterine vein (Oliveira et al., 2008; Bott et al., 2010), leading to the expression of ISG in extra-uterine sites such as the CL (Oliveira et al., 2008), liver (Ruhmann et al., 2017) vaginal and cervical mucosa (Kunii et al., 2018) and peripheral blood leukocytes (Yankey et al., 2001; Han et al., 2006; Kizaki et al., 2013).

While still in the developmental stages with no currently available on farm platform, many research groups aim to increase the application potential of these biomarkers. To date, no assay has been sensitive enough to differentiate peripheral IFNT concentrations between pregnant and non-pregnant females. Several authors, however, have proposed expression of ISG in peripheral blood leukocytes as a possible tool for early pregnancy diagnosis in ruminants (Green et al., 2010; Pugliesi et al., 2014). This technique has been validated in total leukocytes (Han et al., 2006; Stevenson et al., 2007; Green et al., 2010), peripheral blood mononuclear cells (Gifford et al., 2007; Pugliesi et al., 2014; Melo et al., 2020b) and peripheral blood polymorphonuclear cells (Kizaki et al., 2013; Yoshino et al., 2018; Melo et al., 2020a). In general, significant differences between pregnant and non-pregnant females were found between days 18 and 20 of gestation, with accuracy of positive pregnancy diagnosis ranging from 70 to 90% in beef (Pugliesi et al., 2014; Melo et al., 2020a) and dairy herds (Yoshino et al., 2018). Although ISG have been heavily researched as a pregnancy diagnostic tool, high proportions of false-positive and false-negative results significantly reduces this technique’s accuracy. These outcomes could be related to ISG response to other type I interferons, including IFNα and IFNβ, especially in response to viral infections (Schoggins, 2014; Shaw et al., 2017; Shirozu et al., 2017).

In addition to their function as predictors of pregnancy, ISG expression could reflect embryonic viability since there is a positive correlation between the concentrations of IFNT released by the conceptus and the abundance of ISG (Matsuyama et al., 2012). Some studies (Matsuyama et al., 2012; Sheikh et al., 2018) reported different ISG expression patterns between females who maintained pregnancy and females who experienced a pregnancy loss, suggesting that these differences could be explored in the prediction of embryonic or fetal mortality, while others found no differences (Shirasuna et al., 2015; Melo et al., 2020a). Because the events leading to embryonic or fetal mortality are not always related to the development and expansion of the trophectoderm, ISG expression patterns may detect some but not all cases of pregnancy loss. In general, ISG are a valuable tool for pregnancy diagnosis as an indirect stimulus from the conceptus; however, adjustments in techniques are necessary to improve the sensitivity and specificity for potential use in monitoring pregnancy viability.

### Circulating microRNAs

One of the most promising candidates in the search for an easily accessible biomarker for pregnancy diagnosis is circulating microRNAs (miRNA); however, it is currently limited to research use due to the laboratory techniques needed to isolate and measure them. Between 18 to 22 nucleotides in length, miRNAs play important roles in regulation of gene expression and have been found in biological fluids ranging from serum and amniotic fluid to urine and milk (Reid et al., 2011; Pohler et al., 2015). There are many other noncoding RNAs that possess the potential for biomarker discovery in the future; however, miRNAs are currently the best characterized and have been strongly correlated with disease progression and detection (Etheridge et al., 2011; Russo et al., 2016). Furthermore, miRNAs have a well-developed database (miRBase) that includes miRNAs from humans, animals, plants, and viruses (Griffiths-Jones et al., 2008). MicroRNAs have been proposed as optimal biomarkers because they possess key features including stability, non-invasiveness, tissue specificity, and accuracy and rapidity regarding detection methods (Turchinovich et al., 2011; Velu et al., 2012). They have been identified to exist in different forms; miRNAs associated with Argonaute 2 containing protein complexes (Arroyo et al., 2011), lipoproteins (Tabet et al., 2014), or nucleophosmin 1 (Wang et al., 2010), and those located within apoptotic bodies (Zernecke et al., 2009) or extracellular vesicles (Valadi et al., 2007). Extracellular-derived miRNAs are of particular interest as this form of miRNAs remains the most extensively verified, and have been documented to be released by presumably every cell type as a method of cell to cell communication to elicit various biological effects (Turchinovich et al., 2012; Liang et al., 2017). Previous bovine pregnancy-associated miRNA research has resulted in several candidates that were isolated from milk, plasma, serum, or whole blood (Ioannidis and Donadeu, 2016; Bem et al., 2017; Ioannidis and Donadeu, 2017; Pohler et al., 2017; Schanzenbach et al., 2017; Gebremedhn et al., 2018; Markkandan et al., 2018). Studies have also identified miRNAs produced by pregnant animals in horses, sheep and swine (Cameron et al., 2011; Burns et al., 2014; Reliszko et al., 2017). A recent study by Fiandanese et al. (2016) identified a potential miRNA, bta-mir 140, as an early biomarker for pregnancy detection. At day 13, bta-mir 140 was increased in abundance in circulation of pregnant, non-lactating cows and at day 19 it was upregulated in both lactating and non-lactating pregnant cows. In recent studies from our lab, data suggests miRNA may provide additional insight to embryo viability. Cows that experience embryo mortality have a significantly increased abundance of specific miRNAs at days 17 and 24 of gestation compared to cows that have a successful pregnancy (Pohler et al., 2017). Future studies will be needed to assess the repeatability of these findings and to determine precise miRNA most applicable for embryo viability analysis. Additionally, a significant limitation in this area of research is that there is no standardized approach regarding extraction and isolation techniques, which lead to differing results regarding abundance of diverse miRNAs.

## Maternal vs paternal contribution to pregnancy loss

### Female contribution and selection

Regardless of the numerous methods available to identify reproductive failure, selection of females with the innate capacity to carry a pregnancy and produce live offspring is likely the most paramount factor to increase reproductive efficiency. Selection of replacement heifers is often determined by genetics and phenotype; however, certain physiologic characteristics, including assessment of the reproductive tract will increase the likelihood of pregnancy success. Reproductive tract scoring (RTS) has been used to assess the pubertal status of heifers by assessing uterine size and ovarian structures and has been correlated with lifetime productivity of the heifer, especially when evaluated by US (Andersen et al., 1991; Holm et al., 2009). Compared with other single factor pre-breeding variables including age and body weight, RTS was found to have the greatest predictive ability for positive reproductive outcomes including AI pregnancy rate and reduced days to calving (Holm et al., 2009). More recently, antral follicle count has been used in combination with RTS, as high antral follicle count is associated with fewer services per conception (Mossa et al., 2012), increased P4 and estradiol production (Ireland et al., 2009; Jimenez-Krassel et al., 2009), increased uterine protein secretion (McNeel et al., 2017) and greater embryo production (Majumder et al., 2010) contributing to overall improved fertility (Moraes et al., 2019). Within pubertal heifers, however, other physiological differences resulting in sub- and infertility are less apparent. Using a serial ET model to classify beef heifers as high, sub- or infertile, Geary et al. (2016) identified significant alterations in endometrial gene expression between fertility classifications at day 14 of gestation despite similar P4 concentrations and embryo recovery rates, suggesting that significant pregnancy loss occurs between days 14 and 28 in sub- or infertile heifers. These findings were further supported by decreased conceptus length at day 17 of gestation and alterations in endometrial and conceptus transcriptome profiles indicating dysregulation of conceptus-endometrial interactions in sub-fertile heifers compared to high fertility heifers (Moraes et al., 2018). Despite the improvements in replacement heifer selection afforded by ultrasound assessment, not all factors affecting fertility can be as easily identified.

Understanding reproductive failure in cows which have previously been reproductively competent can be specifically challenging. Large reproductive tract size has been associated with decreased fertility in both primiparous and multiparous lactating dairy cows and may be due to sperm transport challenges or increased failure of correct maternal recognition of pregnancy (Baez et al., 2016; Young et al., 2017). The unknown physiological ramifications of increased reproductive tract sizes may persist beyond pregnancy establishment as Madureira et al. (2017) reported increased ovulation failure, decreased circulating concentration of PAGs and increased pregnancy loss between day 30 and 120 in cows with the largest reproductive tracts compared to those with decreased tract sizes.

Another consideration of cow selection, especially when utilizing reproductive technologies, is estrus expression at or prior to insemination. Estrus expression is positively correlated with increased pregnancy rates and decreased embryonic mortality in almost every female classification (parity, subspecies, dairy and beef) undergoing either AI or embryo transfer (Pursley et al., 1998; Richardson et al., 2014; Pereira et al., 2016; Bó and Cedeño, 2018). Numerous aids, including estrus detection patches and activity monitors, have increased the accuracy and effectiveness of estrus detection compared to visual methods which can be subjective and labor intensive. Activity monitors, often mounted on collars or pedometers and relayed to computer software for analysis, have positive predictive values for estrus greater than 85% and daily error rates of less than 2% (Løvendahl and Chagunda, 2010; Madureira et al., 2015). Estrus detection patches, like the Estrotect Breeding Indicator, are single use aids that become activated as animals are repeatedly mounted and require lower input for more extensive management systems (Pohler et al., 2016b). Greater estrus activity, indicated by both patch and activity monitor activation, results in increased day 30 pregnancy rates, greater circulating PAG concentrations and decreased late embryo mortality compared to animals who did not exhibit estrus or only weakly exhibited signs of estrus even when ovulation occurred (Madureira et al., 2015; Pereira et al., 2016; Pohler et al., 2016b). These factors can aid in selection of females with the greatest likelihood for establishing and maintaining pregnancy, although many differences in fertility cannot be yet identified.

### Sire contribution and selection

**Figure 4:**
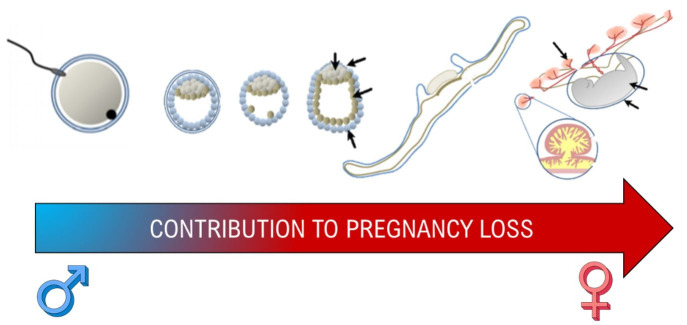
Both female and male contributions are necessary to formation of a successful conceptus but due to the uterine contributions of gestation, the maternal environment is the focus of more research as gestation progresses.

Reproductive physiologists often focus on the female’s role in reproductive processes, and much less attention has been given to male derived factors associated with fertility or causes of embryonic mortality originating from the sire post fertilization and initial embryonic development (Figure 4). In many species, paternal genetics may contribute significantly to placenta formation and subsequent pregnancy maintenance throughout gestation. Using a uniparental embryo model in mice, it was shown that the formation of the embryo is primarily reliant on the maternal genome, while the paternal genome greatly contributes to trophoblast development and, therefore, the placenta (Barton et al., 1985; Surani et al., 1987). In cattle, morphological changes of the placenta associated with placentome formation and interdigitation in intercaruncular spaces begin during the third week of gestation (Schlafer et al., 2000). Adequate placentation is required for proper exchange of nutrients at the fetal-maternal interface and disruption of these physiological processes may lead to pregnancy failure. Research from our lab has characterized the association between sire fertility and pregnancy loss in cattle, as well as began to investigate the physiological mechanisms that paternal genetics control in pregnancy maintenance. In *Bos indicus* beef cows, no difference in pregnancy rate at day 30 was observed among service sires used for AI, but pregnancy loss during the second month of gestation was highly variable (1.4 to 11.1%) among sires (Franco et al., 2018). Similarly, *Bos Taurus* beef cows had a large variance in pregnancy loss between days 24 and 31 of gestation (1.8 to 11.7%) and between days 31 and 60 of gestation (2.3 to 12.6%) among service sires used for AI (Franco et al., 2020). Similarly, same variance was observed among sires used for AI and ET in dairy cattle. Pregnancy loss during the second month of gestation ranged from 5 to 35-40% among sires used in both AI and ET, and no correlation (*P* = 0.8) was observed with their respective sire conception rate (SCR) index (Pohler lab; *Unpublished data*). Interestingly, high SCR sires produce higher quality pre implantation embryos in both *in vivo* and *in vitro* studies, but only discrete differences were observed in embryonic development and pregnancy success in dairy heifers (Ortega et al., 2018).

These sire phenotype characterization studies suggest that current methods to evaluate sire fertility may be limited in assessing overall reproductive success and incidence of late gestation pregnancy loss should be considered when evaluating sire fertility, as it can significantly affect final pregnancy rate. To investigate the physiological mechanism of paternal genetics in pregnancy maintenance during the second month of gestation, parthenogenetic embryos, consisting of only a maternal genome, were transferred into recipient cows at blastocyst stage (day 7) and pregnancy development was monitored by ultrasonography and blood based placental secretions. In all 19 cows that established a pregnancy, circulating concentration of placental products (PAG and ISG) throughout gestation were lesser compared to cows carrying control embryos (Pohler Lab; *Unpublished data*). Preliminary results demonstrate that even though these embryos can survive up to day 40-45 of gestation, no active site of implantation and attachment to endometrium is observed, suggesting that trophoblast tissues are not properly formed in the absence of paternal genes. These findings strongly suggest that paternal genetics contribute significantly to placenta formation in cattle, which could explain most of the sire variance observed in pregnancy loss during the period of active placentation. The development of markers to identify sires of high or low pregnancy loss would improve sire fertility evaluations and increase beef and dairy reproductive efficiency.

## Summary

Reproductive failure, embryonic *mortality* and pregnancy loss are major challenges facing domestic livestock producers worldwide. In science, we must develop technologies and management strategies to decrease these losses. These include managerial and nutritional tools that promote embryonic and fetal development, resulting in a successful pregnancy (Cooke, 2019). However, in order to do this, technologies and techniques for predicting, forecasting and determining pregnancy success and failure must continue to be developed. Many current technologies (Figure 2) are useful, but there is still a demand to increase our detection systems, specifically early in gestation.

## References

[B001] Andersen K, LeFever D, Brinks J, Odde K (1991). The use of reproductive tract scoring in beef heifers. Agri-Practice..

[B002] Arnold H, Martins JP, Oliveira LZ, Policelli R, Stomack K, Sasser G, Pursley J (2012). Effectiveness of pregnancy-specific protein B in pregnancy diagnosis of dairy cows and heifers. J Dairy Sci.

[B003] Arroyo JD, Chevillet JR, Kroh EM, Ruf IK, Pritchard CC, Gibson DF, Mitchell PS, Bennett CF, Pogosova-Agadjanyan EL, Stirewalt DL, Tait JF, Tewari M (2011). Argonaute2 complexes carry a population of circulating microRNAs independent of vesicles in human plasma. Proc Natl Acad Sci USA.

[B004] Assis AC, Pereira F, Santos TC, Ambrosio CE, Leiser R, Miglino MA (2010). Morpho‐physical recording of bovine conceptus (Bos indicus) and placenta from days 20 to 70 of pregnancy. Reprod Domest Anim.

[B005] Austin KJ, King CP, Vierk JE, Sasser RG, Hansen TR (1999). Pregnancy-specific protein B induces release of an alpha chemokine in bovine endometrium. Endocrinology.

[B006] Baez GM, Barletta RV, Guenther JN, Gaska JM, Wiltbank MC (2016). Effect of uterine size on fertility of lactating dairy cows. Theriogenology.

[B007] Balaro MFA, Santos AS, Moura LFG, Fonseca JF, Brandão FZ (2017). Luteal dynamic and functionality assessment in dairy goats by luteal blood flow, luteal biometry, and hormonal assay. Theriogenology.

[B008] Ball L, Carroll E (1963). Induction of fetal death in cattle by manual rupture of the amniotic vesicle. J Am Vet Med Assoc.

[B009] Barton SC, Adams CA, Norris M, Surani M (1985). Development of gynogenetic and parthenogenetic inner cell mass and trophectoderm tissues in reconstituted blastocysts in the mouse. J Embryol Exp Morphol.

[B010] Bazer FW, Spencer TE, Ott TL (1997). Interferon tau: a novel pregnancy recognition signal. Am J Reprod Immunol.

[B011] Bazer FW, Ying W, Wang X, Dunlap KA, Zhou B, Johnson GA, Wu G (2015). The many faces of interferon tau. Amino Acids.

[B012] Beal WE, Perry RC, Corah LR (1992). The use of ultrasound in monitoring reproductive physiology of beef cattle. J Anim Sci.

[B013] Bem THC, Silveira JC, Sampaio RV, Sangalli JR, Oliveira MLF, Ferreira RM, Silva LA, Perecin F, King WA, Meirelles FV, Ramos ES (2017). Low levels of exosomal-miRNAs in maternal blood are associated with early pregnancy loss in cloned cattle. Sci Rep.

[B014] Bó GA, Cedeño A (2018). Expression of estrus as a relevant factor in fixed-time embryo transfer programs using estradiol/progesterone-based protocols in cattle. Anim Reprod.

[B015] Bott RC, Ashley RL, Henkes LE, Antoniazzi AQ, Bruemmer JE, Niswender GD, Bazer FW, Spencer TE, Smirnova NP, Anthony RV, Hansen TR (2010). Uterine vein infusion of interferon tau (IFNT) extends luteal life span in ewes. Biol Reprod.

[B016] Breukelman S, Perényi Z, Taverne M, Jonker H, Van der Weijden G, Vos P, de Ruigh L, Dieleman S, Beckers J-F, Szenci O (2012). Characterisation of pregnancy losses after embryo transfer by measuring plasma progesterone and bovine pregnancy-associated glycoprotein-1 concentrations. Vet J.

[B017] Burns G, Brooks K, Wildung M, Navakanitworakul R, Christenson LK, Spencer TE (2014). Extracellular vesicles in luminal fluid of the ovine uterus. PLoS One.

[B018] Butler J, Hamilton W, Sasser R, Ruder C, Hass G, Williams R (1982). Detection and partial characterization of two bovine pregnancy-specific proteins. Biol Reprod.

[B019] Cameron A, Silveira J, Bouma G, Bruemmer J (2011). Evaluation of exosomes containing miRNA as an indicator of pregnancy status in the mare. J Equine Vet Sci.

[B020] Carter F, Forde N, Duffy P, Wade M, Fair T, Crowe M, Evans A, Kenny D, Roche J, Lonergan P (2008). Effect of increasing progesterone concentration from Day 3 of pregnancy on subsequent embryo survival and development in beef heifers. Reprod Fertil Dev.

[B021] Cooke RF (2019). Early career achievement award: supplementing omega-6 fatty acids to enhance early embryonic development and pregnancy establishment in Bos indicus and B. taurus beef cows. J Anim Sci.

[B022] Curran S, Pierson R, Ginther O (1986). Ultrasonographic appearance of the bovine conceptus from days 20 through 60. J Am Vet Med Assoc.

[B023] Diskin M, Sreenan J (1980). Fertilization and embryonic mortality rates in beef heifers after artificial insemination. J Reprod Fertil.

[B024] Etheridge A, Lee I, Hood L, Galas D, Wang K (2011). Extracellular microRNA: a new source of biomarkers. Mutat Res.

[B025] Fiandanese N, Viglino A, Strozzi F, Stella A, Williams JL, Lonergan P, Forde N, Iamartino D (2016). 71 Circulating microRNAs as potential biomarkers of early pregnancy in high producing dairy cows. Reprod Fertil Dev.

[B026] Franco G, Reese S, Poole R, Rhinehart J, Thompson K, Cooke R, Pohler K (2020). Sire contribution to pregnancy loss in different periods of embryonic and fetal development of beef cows. Theriogenology.

[B027] Franco GA, Peres RFG, Martins CFG, Reese ST, Vasconcelos JLM, Pohler KG (2018). Sire contribution to pregnancy loss and pregnancy-associated glycoprotein production in Nelore cows. J Anim Sci.

[B028] Garrett J, Geisert R, Zavy M, Morgan G (1988). Evidence for maternal regulation of early conceptus growth and development in beef cattle. J Reprod Fertil.

[B029] Gatea AO, Smith MF, Pohler KG, Egen T, Pereira MH, Vasconselos JL, Lawrence JC, Green JA (2018). The ability to predict pregnancy loss in cattle with ELISAs that detect pregnancy associated glycoproteins is antibody dependent. Theriogenology.

[B030] Geary TW, Burns GW, Moraes JG, Moss JI, Denicol AC, Dobbs KB, Ortega MS, Hansen PJ, Wehrman ME, Neibergs H, O’Neil E, Behura S, Spencer TE (2016). Identification of beef heifers with superior uterine capacity for pregnancy 1. Biol Reprod.

[B031] Gebremedhn S, Salilew-Wondim D, Hoelker M, Held-Hoelker E, Neuhoff C, Tholen E, Schellander K, Tesfaye D (2018). Exploring maternal serum microRNAs during early pregnancy in cattle. Theriogenology.

[B032] Gifford C, Racicot K, Clark D, Austin K, Hansen T, Lucy M, Davies C, Ott T (2007). Regulation of interferon-stimulated genes in peripheral blood leukocytes in pregnant and bred, nonpregnant dairy cows. J Dairy Sci.

[B033] Ginther O, Shrestha H, Fuenzalida M, Shahiduzzaman A, Beg M (2010). Characteristics of pulses of 13, 14-dihydro-15-keto-prostaglandin F2alpha before, during, and after spontaneous luteolysis and temporal intrapulse relationships with progesterone concentrations in cattle. Biol Reprod.

[B034] Gray C, Burghardt R, Johnson G, Bazer F, Spencer T (2002). Evidence that absence of endometrial gland secretions in uterine gland knockout ewes compromises conceptus survival and elongation. Reproduction.

[B035] Green J, Okamura C, Poock S, Lucy M (2010). Measurement of interferon-tau (IFN-τ) stimulated gene expression in blood leukocytes for pregnancy diagnosis within 18-20d after insemination in dairy cattle. Anim Reprod Sci.

[B036] Griffiths-Jones S, Saini HK, van Dongen S, Enright AJ (2008). miRBase: tools for microRNA genomics. Nucleic Acids Res.

[B037] Han H, Austin KJ, Rempel LA, Hansen TR (2006). Low blood ISG15 mRNA and progesterone levels are predictive of non-pregnant dairy cows. J Endocrinol.

[B038] Hata T, Aoki S, Manabe A, Hata K, Miyazaki K (1997). Three-dimensional ultrasonography in the first trimester of human pregnancy. Hum Reprod.

[B039] Herzog K, Brockhan-Lüdemann M, Kaske M, Beindorff N, Paul V, Niemann H, Bollwein H (2010). Luteal blood flow is a more appropriate indicator for luteal function during the bovine estrous cycle than luteal size. Theriogenology.

[B040] Hoeben D, Burvenich C, Massart-Leën A-M, Lenjou M, Nijs G, Van Bockstaele D, Beckers J-F (1999). In vitro effect of ketone bodies, glucocorticosteroids and bovine pregnancy-associated glycoprotein on cultures of bone marrow progenitor cells of cows and calves. Vet Immunol Immunopathol.

[B041] Holm DE, Thompson PN, Irons PC (2009). The value of reproductive tract scoring as a predictor of fertility and production outcomes in beef heifers. J Anim Sci.

[B042] Ioannidis J, Donadeu FX (2016). Circulating miRNA signatures of early pregnancy in cattle. BMC Genomics.

[B043] Ioannidis J, Donadeu FX (2017). Changes in circulating microRNA levels can be identified as early as day 8 of pregnancy in cattle. PLoS One.

[B044] Ireland J, Zielak-Steciwko A, Jimenez-Krassel F, Folger J, Bettegowda A, Scheetz D, Walsh S, Mossa F, Knight P, Smith G, Lonergan P, Evans AC (2009). Variation in the ovarian reserve is linked to alterations in intrafollicular estradiol production and ovarian biomarkers of follicular differentiation and oocyte quality in cattle. Biol Reprod.

[B045] Jimenez-Krassel F, Folger J, Ireland J, Smith G, Hou X, Davis JS, Lonergan P, Evans A, Ireland J (2009). Evidence that high variation in ovarian reserves of healthy young adults has a negative impact on the corpus luteum and endometrium during estrous cycles in cattle. Biol Reprod.

[B046] Kähn W (1989). Sonographic fetometry in the bovine. Theriogenology.

[B047] Kastelic J, Curran S, Pierson R, Ginther O (1988). Ultrasonic evaluation of the bovine conceptus. Theriogenology.

[B048] Kizaki K, Shichijo-Kizaki A, Furusawa T, Takahashi T, Hosoe M, Hashizume K (2013). Differential neutrophil gene expression in early bovine pregnancy. Reprod Biol Endocrinol.

[B049] Kunii H, Koyama K, Ito T, Suzuki T, Balboula AZ, Shirozu T, Bai H, Nagano M, Kawahara M, Takahashi M (2018). Hot topic: pregnancy-induced expression of interferon-stimulated genes in the cervical and vaginal mucosal membranes. J Dairy Sci.

[B050] Liang J, Wang S, Wang Z (2017). Role of microRNAs in embryo implantation. Reprod Biol Endocrinol.

[B051] Løvendahl P, Chagunda M (2010). On the use of physical activity monitoring for estrus detection in dairy cows. J Dairy Sci.

[B052] Lucy M (2001). Reproductive loss in high-producing dairy cattle: where will it end?. J Dairy Sci.

[B053] Madureira A, Silper B, Burnett T, Polsky L, Cruppe L, Veira D, Vasconcelos J, Cerri R (2015). Factors affecting expression of estrus measured by activity monitors and conception risk of lactating dairy cows. J Dairy Sci.

[B054] Madureira AML, Franco GA, Guida TG, Edwards JL, Schrick FN, Vasconcelos JLM, Cerri RLA, Pohler KG (2017). Effect of size and position of the reproductive tract on concentrations of bovine Pregnancy Associated Glycoproteins (PAGs) and the relationship with fertility.

[B055] Majumder K, Gelbaya TA, Laing I, Nardo LG (2010). The use of anti-Müllerian hormone and antral follicle count to predict the potential of oocytes and embryos. Eur J Obstet Gynecol Reprod Biol.

[B056] Mann G, Lamming G (1999). The influence of progesterone during early pregnancy in cattle. Reprod Domest Anim.

[B057] Markkandan K, Ahn K, Lee DJ, Kim TI, Dang C, Hong S-E, Yoon H-B, Lim H-J, Hong CP (2018). Profiling and identification of pregnancy-associated circulating microRNAs in dairy cattle. Genes Genomics.

[B058] Martins J, Wang D, Mu N, Rossi G, Martini A, Martins V, Pursley J (2018). Level of circulating concentrations of progesterone during ovulatory follicle development affects timing of pregnancy loss in lactating dairy cows. J Dairy Sci.

[B059] Matsuyama S, Kojima T, Kato S, Kimura K (2012). Relationship between quantity of IFNT estimated by IFN-stimulated gene expression in peripheral blood mononuclear cells and bovine embryonic mortality after AI or ET. Reprod Biol Endocrinol.

[B060] McNeel AK, Soares ÉM, Patterson AL, Vallet JL, Wright EC, Larimore EL, Amundson OL, Miles JR, Chase CC, Lents CA, Wood JR, Cupp AS, Perry GA, Cushman RA (2017). Beef heifers with diminished numbers of antral follicles have decreased uterine protein concentrations. Anim Reprod Sci.

[B061] Melo GD, Mello BP, Ferreira CA, Godoy CAS, Rocha CC, Silva AG, Reese ST, Madureira EH, Pohler KG, Pugliesi G (2020). Applied use of interferon-tau stimulated genes expression in polymorphonquclear cells to detect pregnancy compared to other early predictors in beef cattle. Theriogenology.

[B062] Melo GD, Pinto LFM, Rocha CC, Motta IG, Silva LA, Silveira JC, Gonella-Diaza AM, Binelli M, Pugliesi G (2020). Type I interferon receptors and interferon-t stimulated genes in peripheral blood mononuclear cells and polymorphonuclear leucocytes in early pregnancy in beef heifers. Reprod Fertil Dev.

[B063] Middleton E, Pursley J (2019). Blood samples before and after embryonic attachment accurately determine non-pregnant lactating dairy cows at 24 d post-artificial insemination using a commercially available assay for pregnancy-specific protein B. J Dairy Sci.

[B064] Mirando M, Short E, Geisert R, Vallet J, Bazer F (1991). Stimulation of 2′, 5′-oligoadenylate synthetase activity in sheep endometrium during pregnancy, by intrauterine infusion of ovine trophoblast protein-1, and by intramuscular administration of recombinant bovine interferon-αI1. J Reprod Fertil.

[B065] Momont H (1990). Rectal palpation: safety issues.

[B066] Moraes FLZ, Morotti F, Costa CB, Lunardelli PA, Seneda MM (2019). Relationships between antral follicle count, body condition, and pregnancy rates after timed-AI in Bos indicus cattle. Theriogenology.

[B067] Moraes JG, Behura SK, Geary TW, Hansen PJ, Neibergs HL, Spencer TE (2018). Uterine influences on conceptus development in fertility-classified animals. Proc Natl Acad Sci USA.

[B068] Mossa F, Walsh S, Butler S, Berry D, Carter F, Lonergan P, Smith G, Ireland J, Evans A (2012). Low numbers of ovarian follicles≥ 3 mm in diameter are associated with low fertility in dairy cows. J Dairy Sci.

[B069] Motta IG, Rocha CC, Bisinotto DZ, Melo GD, Ataide GA, Silva AG, Gonzaga VHG, Santos JA, Freitas BG, Lemes KM, Madureira EH (2020). Increased pregnancy rate in beef heifers resynchronized with estradiol at 14 days after TAI. Theriogenology.

[B070] Nebel RL, Whittier W, Cassell B, Britt J (1987). Comparison of on-farm and laboratory milk progesterone assays for identifying errors in detection of estrus and diagnosis of pregnancy. J Dairy Sci.

[B071] Nebel R (1988). On-farm milk progesterone tests. J Dairy Sci.

[B072] Niswender GD, Juengel JL, Silva PJ, Rollyson MK, McIntush EW (2000). Mechanisms controlling the function and life span of the corpus luteum. Physiol Rev.

[B073] Oliveira R, Franco G, Reese S, Dantas F, Fontes P, Cooke R, Rhinehart J, Thompson K, Pohler K (2020). Using pregnancy associated glycoproteins (PAG) for pregnancy detection at day 24 of gestation in beef cattle. Theriogenology.

[B074] Oliveira JF, Henkes LE, Ashley RL, Purcell SH, Smirnova NP, Veeramachaneni DR, Anthony RV, Hansen TR (2008). Expression of interferon (IFN)-stimulated genes in extrauterine tissues during early pregnancy in sheep is the consequence of endocrine IFN-τ release from the uterine vein. Endocrinology.

[B075] Omontese B, Gomes G, Santos A, Silva L, Merenda V, Bisinotto R (2020). Use of on-farm milk progesterone information to predict fertility outcomes in dairy cows subjected to timed artificial insemination. J Dairy Sci.

[B076] Ortega MS, Moraes JG, Patterson DJ, Smith MF, Behura SK, Poock S, Spencer TE (2018). Influences of sire conception rate on pregnancy establishment in dairy cattle. Biol Reprod.

[B077] Parr M, Mullen M, Crowe M, Roche J, Lonergan P, Evans A, Diskin M (2012). Relationship between pregnancy per artificial insemination and early luteal concentrations of progesterone and establishment of repeatability estimates for these traits in Holstein-Friesian heifers. J Dairy Sci.

[B078] Pereira M, Wiltbank M, Vasconcelos J (2016). Expression of estrus improves fertility and decreases pregnancy losses in lactating dairy cows that receive artificial insemination or embryo transfer. J Dairy Sci.

[B079] Pierson R, Ginther O (1984). Ultrasonography for detection of pregnancy and study of embryonic development in heifers. Theriogenology.

[B080] Pohler KG, Geary TW, Johnson CL, Atkins JA, Jinks EM, Busch DC, Green JA, MacNeil MD, Smith MF (2013). Circulating bovine pregnancy associated glycoproteins are associated with late embryonic/fetal survival but not ovulatory follicle size in suckled beef cows. J Anim Sci.

[B081] Pohler KG, Green JA, Geary TW, Peres RF, Pereira MH, Vasconcelos JL, Smith MF (2015). Predicting embryo presence and viability. Adv Anat Embryol Cell Biol.

[B082] Pohler KG, Green JA, Moley LA, Doran KM, Graff HB, Peres RFG, Vasconcelos JLM, Smith MF (2014). The effect of embryonic size and sire on circulating concentrations of bovine pregnancy associated glycoproteins in beef cattle..

[B083] Pohler KG, Green JA, Moley LA, Gunewardena S, Hung W-T, Payton RR, Hong X, Christenson LK, Geary TW, Smith MF (2017). Circulating microRNA as candidates for early embryonic viability in cattle. Mol Reprod Dev.

[B084] Pohler KG, Pereira MHC, Lopes FR, Lawrence JC, Keisler DH, Smith MF, Vasconcelos JLM, Green JA (2016). Circulating concentrations of bovine pregnancy-associated glycoproteins and late embryonic mortality in lactating dairy herds. J Dairy Sci.

[B085] Pohler KG, Peres RFG, Green JA, Graff H, Martins T, Vasconcelos JLM, Smith MF (2016). Use of bovine pregnancy-associated glycoproteins to predict late embryonic mortality in postpartum Nelore beef cows. Theriogenology.

[B086] Pope W (1988). Uterine asynchrony: a cause of embryonic loss. Biol Reprod.

[B087] Pugliesi G, de Melo GD, Ataíde GA, Pellegrino CAG, Silva JB, Rocha CC, Motta IG, Vasconcelos JLM, Binelli M (2018). Use of Doppler ultrasonography in embryo transfer programs: feasibility and field results. Animal Reproduction (AR).

[B088] Pugliesi G, Miagawa BT, Paiva YN, França MR, Silva LA, Binelli M (2014). Conceptus-induced changes in the gene expression of blood immune cells and the ultrasound-accessed luteal function in beef cattle: how early can we detect pregnancy?. Biol Reprod.

[B089] Pursley JR, Silcox RW, Wiltbank MC (1998). Effect of time of artificial insemination on pregnancy rates, calving rates, pregnancy loss, and gender ratio after synchronization of ovulation in lactating dairy cows. J Dairy Sci.

[B090] Reese ST, Franco GA, Poole RK, Hood R, Fernadez Montero L, Oliveira RV, Cooke RF, Pohler KG (2020). Pregnancy loss in beef cattle: a meta-analysis. Anim Reprod Sci.

[B091] Reese S, Geary T, Franco G, Moraes J, Spencer T, Pohler K (2019). Pregnancy associated glycoproteins (PAGs) and pregnancy loss in high vs sub fertility heifers. Theriogenology.

[B092] Reese ST, Pereira MHC, Edwards JL, Vasconcelos JLM, Pohler KG (2018). Pregnancy diagnosis in cattle using pregnancy associated glycoprotein concentration in circulation at day 24 of gestation. Theriogenology.

[B093] Reid G, Kirschner MB, van Zandwijk N (2011). Circulating microRNAs: association with disease and potential use as biomarkers. Crit Rev Oncol Hematol.

[B094] Reliszko ZP, Gajewski Z, Kaczmarek MM (2017). Signs of embryo-maternal communication: miRNAs in the maternal serum of pregnant pigs. Reproduction.

[B095] Richardson B, Hill S, Stevenson J, Djira J, Perry G (2014). Meta-analysis of the effect of estrus expression before fixed-time AI on conception rates in beef cattle. J Anim Sci.

[B096] Roberts SJ (1956). Veterinary obstetrics and genital diseases.

[B097] Roche J, Bolandl M, McGeady T (1981). Reproductive wastage following artificial insemination of heifers. Vet Rec.

[B098] Romano JE, Larson JE (2010). Accuracy of pregnancy specific protein-B test for early pregnancy diagnosis in dairy cattle. Theriogenology.

[B099] Romano JE, Thompson JA, Forrest DW, Westhusin ME, Tomaszweski MA, Kraemer DC (2006). Early pregnancy diagnosis by transrectal ultrasonography in dairy cattle. Theriogenology.

[B100] Ruhmann B, Giller K, Hankele A-K, Ulbrich S, Schmicke M (2017). Interferon-τ induced gene expression in bovine hepatocytes during early pregnancy. Theriogenology.

[B101] Russo F, Scoyni F, Fatica A, Pellegrini M, Ferro A, Pulvirenti A, Giugno R, García-Giménez JL (2016). Circulating noncoding RNAs as clinical biomarkers. Epigenetic biomarkers and diagnostics.

[B102] Santos J, Thatcher W, Chebel R, Cerri R, Galvão K (2004). The effect of embryonic death rates in cattle on the efficacy of estrus synchronization programs. Anim Reprod Sci.

[B103] Sasser R, Ruder C (1987). Detection of early pregnancy in domestic ruminants. J Reprod Fertil Suppl.

[B104] Schanzenbach CI, Kirchner B, Ulbrich SE, Pfaffl MW (2017). Can milk cell or skim milk miRNAs be used as biomarkers for early pregnancy detection in cattle?. PLoS One.

[B105] Schlafer D, Fisher P, Davies C (2000). The bovine placenta before and after birth: placental development and function in health and disease. Anim Reprod Sci.

[B106] Schoggins JW (2014). Interferon-stimulated genes: roles in viral pathogenesis. Curr Opin Virol.

[B107] Scully S, Evans A, Carter F, Duffy P, Lonergan P, Crowe M (2015). Ultrasound monitoring of blood flow and echotexture of the corpus luteum and uterus during early pregnancy of beef heifers. Theriogenology.

[B108] Shaw AE, Hughes J, Gu Q, Behdenna A, Singer JB, Dennis T, Orton RJ, Varela M, Gifford RJ, Wilson SJ, Palmarini M (2017). Fundamental properties of the mammalian innate immune system revealed by multispecies comparison of type I interferon responses. PLoS Biol.

[B109] Sheikh AA, Hooda O, Kalyan A, Kamboj A, Mohammed S, Alhussien M, Reddi S, Shimray PG, Rautela A, Pandita S, Kapila S, De S, Dang AK (2018). Interferon-tau stimulated gene expression: A proxy to predict embryonic mortality in dairy cows. Theriogenology.

[B110] Shirasuna K, Matsumoto H, Matsuyama S, Kimura K, Bollwein H, Miyamoto A (2015). Possible role of IFNT on the bovine corpus luteum and neutrophils during the early pregnancy. Reproduction.

[B111] Shirozu T, Iwano H, Ogiso T, Suzuki T, Balboula AZ, Bai H, Kawahara M, Kimura K, Takahashi H, Rulan B, Kim SW, Yanagawa Y, Nagano M, Imakawa K, Takahashi M (2017). Estrous cycle stage-dependent manner of type I interferon-stimulated genes induction in the bovine endometrium. J Reprod Dev.

[B112] Silva E, Sterry R, Kolb D, Mathialagan N, McGrath M, Ballam J, Fricke P (2007). Accuracy of a pregnancy-associated glycoprotein ELISA to determine pregnancy status of lactating dairy cows twenty-seven days after timed artificial insemination. J Dairy Sci.

[B113] Siqueira L, Areas V, Ghetti A, Fonseca J, Palhao M, Fernandes C, Viana J (2013). Color Doppler flow imaging for the early detection of nonpregnant cattle at 20 days after timed artificial insemination. J Dairy Sci.

[B114] Sousa N, Ayad A, Beckers J, Gajewski Z (2006). Pregnancy-associated glycoproteins (PAG) as pregnancy markers in the ruminants. J Physiol Pharmacol.

[B115] Starbuck MJ, Dailey RA, Inskeep EK (2004). Factors affecting retention of early pregnancy in dairy cattle. Anim Reprod Sci.

[B116] Stevenson J, Dalton J, Ott T, Racicot K, Chebel R (2007). Correlation between reproductive status and steady-state messenger ribonucleic acid levels of the resistance gene, MX2, in peripheral blood leukocytes of dairy heifers. J Anim Sci.

[B117] Stronge A, Sreenan J, Diskin M, Mee J, Kenny D, Morris D (2005). Post-insemination milk progesterone concentration and embryo survival in dairy cows. Theriogenology.

[B118] Surani M, Barton S, Norris M (1987). Influence of parental chromosomes on spatial specificity in androgenetic↔ parthenogenetic chimaeras in the mouse. Nature.

[B119] Szenci O, Humblot P, Beckers J, Sasser G, Sulon J, Baltusen R, Varga J, Bajcsy CÁ, Taverne M (2000). Plasma profiles of progesterone and conceptus proteins in cows with spontaneous embryonic/fetal mortality as diagnosed by ultrasonography. Vet J.

[B120] Tabet F, Vickers KC, Torres LFC, Wiese CB, Shoucri BM, Lambert G, Catherinet C, Prado-Lourenco L, Levin MG, Thacker S (2014). HDL-transferred microRNA-223 regulates ICAM-1 expression in endothelial cells. Nat Commun.

[B121] Thompson I, Cerri R, Kim I, Green J, Santos J, Thatcher W (2010). Effects of resynchronization programs on pregnancy per artificial insemination, progesterone, and pregnancy-associated glycoproteins in plasma of lactating dairy cows. J Dairy Sci.

[B122] Turchinovich A, Weiz L, Burwinkel B (2012). Extracellular miRNAs: the mystery of their origin and function. Trends Biochem Sci.

[B123] Turchinovich A, Weiz L, Langheinz A, Burwinkel B (2011). Characterization of extracellular circulating microRNA. Nucleic Acids Res.

[B124] USDA (2009). NAHMS dairy 2007 part IV: reference of dairy cattle health and management practices in the United States.

[B125] USDA (2010). NAHMS Beef 2007-2008.

[B126] Valadi H, Ekström K, Bossios A, Sjöstrand M, Lee JJ, Lötvall JO (2007). Exosome-mediated transfer of mRNAs and microRNAs is a novel mechanism of genetic exchange between cells. Nat Cell Biol.

[B127] Velu VK, Ramesh R, Srinivasan A (2012). Circulating microRNAs as biomarkers in health and disease. J Clin Diagn Res.

[B128] Wallace RM, Pohler KG, Smith MF, Green JA (2015). Placental PAGs: gene origins, expression patterns, and use as markers of pregnancy. Reproduction.

[B129] Wang K, Zhang S, Weber J, Baxter D, Galas DJ (2010). Export of microRNAs and microRNA-protective protein by mammalian cells. Nucleic Acids Res.

[B130] Wiltbank MC, Baez GM, Garcia-Guerra A, Toledo MZ, Monteiro PL, Melo LF, Ochoa JC, Santos JE, Sartori R (2016). Pivotal periods for pregnancy loss during the first trimester of gestation in lactating dairy cows. Theriogenology.

[B131] Wooding F, Wathes DC (1980). Binucleate cell migration in the bovine placentome. J Reprod Fertil.

[B132] Wooding F (1982). Structure and function of placental binucleate (giant) cells. Bibl Anat.

[B133] Yankey S, Hicks B, Carnahan K, Assiri A, Sinor S, Kodali K, Stellflug J, Ott T (2001). Expression of the antiviral protein Mx in peripheral blood mononuclear cells of pregnant and bred, non-pregnant ewes. J Endocrinol.

[B134] Yoshino H, Toji N, Sasaki K, Koshi K, Yamagishi N, Takahashi T, Ishiguro-Oonuma T, Matsuda H, Yamanouchi T, Hashiyada Y, Imai K, Izaike Y, Kizaki K, Hashizume K (2018). A predictive threshold value for the diagnosis of early pregnancy in cows using interferon-stimulated genes in granulocytes. Theriogenology.

[B135] Young C, Schrick F, Pohler K, Saxton A, Di Croce F, Roper D, Wilkerson J, Edwards J (2017). A reproductive tract scoring system to manage fertility in lactating dairy cows. J Dairy Sci.

[B136] Zernecke A, Bidzhekov K, Noels H, Shagdarsuren E, Gan L, Denecke B, Hristov M, Köppel T, Jahantigh MN, Lutgens E, Wang S, Olson EN, Schober A, Weber C (2009). Delivery of microRNA-126 by apoptotic bodies induces CXCL12-dependent vascular protection. Sci Signal.

