# Erratum

**DOI:** 10.1590/1984-3143-AR2021-0033

**Published:** 2021-06-18

In the article entitle “New approaches to diagnose and target reproductive failure in cattle”, DOI number: http://dx.doi.org/10.1590/1984-3143-ar2020-0057, published in journal Animal Reproduction, 2020, vol.17, number 3, p. 10:

Where it reads:

Reproductive physiologists often focus on the female’s role in reproductive processes, and much less attention has been given to male derived factors associated with fertility or causes of embryonic mortality originating from the sire post fertilization and initial embryonic development (Figure 4)

It should read:

Reproductive physiologists often focus on the female’s role in reproductive processes, and much less attention has been given to male derived factors associated with fertility or causes of embryonic mortality originating from the sire post fertilization and initial embryonic development (Figure 4 - Bai et al., 2013).

Where it reads:

**Figure 4:**
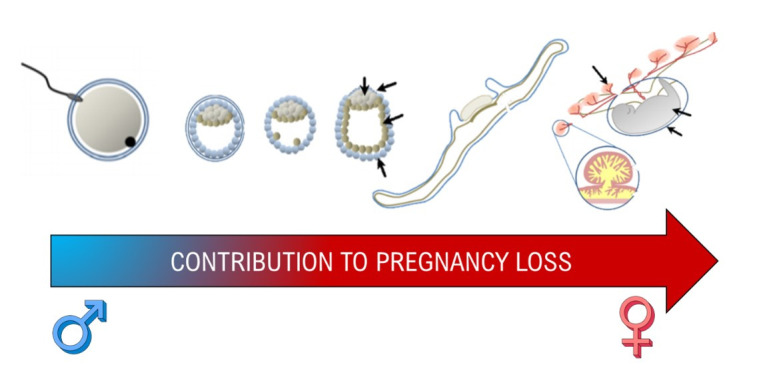
Both female and male contributions are necessary to formation of a successful conceptus but due to the uterine contributions of gestation, the maternal environment is the focus of more research as gestation progresses.

It should read:

**Figure 4:**
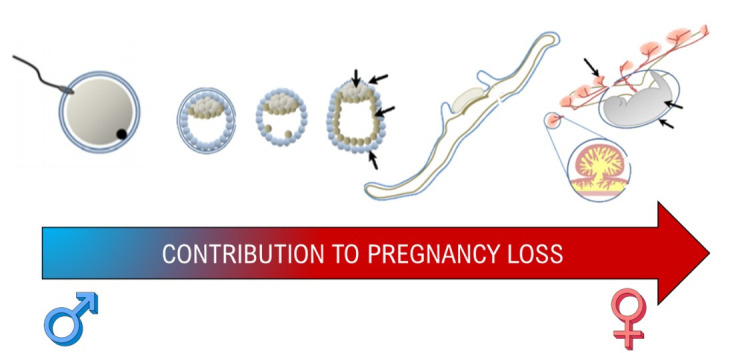
Both female and male contributions are necessary to formation of a successful conceptus but due to the uterine contributions of gestation, the maternal environment is the focus of more research as gestation progresses (Bai et al., 2013).

Where it reads:

Baez GM, Barletta RV, Guenther JN, Gaska JM, Wiltbank MC. Effect of uterine size on fertility of lactating dairy cows. Theriogenology. 2016;85(8):1357-66. http://dx.doi.org/10.1016/j.theriogenology.2015.04.022. PMid:26924681.

Balaro MFA, Santos AS, Moura LFG, Fonseca JF, Brandão FZ. Luteal dynamic and functionality assessment in dairy goats by luteal blood flow, luteal biometry, and hormonal assay. Theriogenology. 2017;95:118-26. http://dx.doi.org/10.1016/j.theriogenology.2017.02.021. PMid:28460665.

It should read:

Baez GM, Barletta RV, Guenther JN, Gaska JM, Wiltbank MC. Effect of uterine size on fertility of lactating dairy cows. Theriogenology. 2016;85(8):1357-66. http://dx.doi.org/10.1016/j.theriogenology.2015.04.022. PMid:26924681.

Bai H, Sakurai T, Godkin JD, Imakawa K. Expression and potential role of GATA factors in trophoblast development. J Reprod Dev. 2013;59:1– 6. https://doi.org/10.1262/jrd.2012-100. PMid:23428586

Balaro MFA, Santos AS, Moura LFG, Fonseca JF, Brandão FZ. Luteal dynamic and functionality assessment in dairy goats by luteal blood flow, luteal biometry, and hormonal assay. Theriogenology. 2017;95:118-26. http://dx.doi.org/10.1016/j.theriogenology.2017.02.021. PMid:28460665.

